# Development of a Patient-Reported Outcome Instrument for Patients With Severe Lower Extremity Trauma (LIMB-Q): Protocol for a Multiphase Mixed Methods Study

**DOI:** 10.2196/14397

**Published:** 2019-10-17

**Authors:** Lily R Mundy, Anne Klassen, Jordan Grier, Matthew J Carty, Andrea L Pusic, Scott T Hollenbeck, Mark J Gage

**Affiliations:** 1 Division of Plastic and Reconstructive Surgery Duke University Durham, NC United States; 2 Department of Pediatrics McMaster University Hamilton, ON Canada; 3 Section of Orthopaedic Trauma Department of Orthopaedic Surgery Duke University Durham, NC United States; 4 Division of Plastic and Reconstructive Surgery Brigham and Women’s Hospital Boston, MA United States; 5 Patient Reported Outcomes, Value & Experience Center Brigham and Women’s Hospital Boston, MA United States

**Keywords:** amputation, limb salvage, lower extremity, trauma, survey, questionnaire, patient reported outcome measures, quality of life, patient satisfaction

## Abstract

**Background:**

A current limitation in the care of patients with severe lower extremity traumatic injuries is the lack of a rigorously developed patient-reported outcome (PRO) instrument specific to lower extremity trauma patients.

**Objective:**

This mixed methods protocol aims to describe phases I and II of the development of a PRO instrument for lower extremity trauma patients, following international PRO development guidelines.

**Methods:**

The phase I study follows an interpretive description approach. Development of the PRO instrument begins with identifying the concepts that are important to patients, after which a preliminary conceptual framework is devised from a systematic literature review and used to generate an interview guide. Patients aged 18 years or above with limb-threatening lower extremity traumatic injuries resulting in reconstruction, amputation, or amputation after failed reconstruction will be recruited. The subjects will participate in semistructured, in-depth qualitative interviews to identify all important concepts of interest. The qualitative interview data will be coded with top-level domains, themes, and subthemes. The codes will then be utilized to refine the conceptual framework and generate preliminary items and a set of scales. The preliminary scales will be further refined via a process of conducting cognitive debriefing interviews with lower extremity trauma patients and soliciting expert opinions. Phase III will include a large-scale field test, using Rasch measurement theory to analyze the psychometric properties of the instrument; shortening and finalizing the scales; and determining the reliability, validity, and responsiveness of the instrument.

**Results:**

Phases I and II of this study have been funded. Phase I of this study has been completed, and phase II began in January 2019 and is expected to be completed in November 2019. Phase III will begin following the completion of phase II.

**Conclusions:**

This protocol describes the initial phases of development of a novel PRO instrument for use in lower extremity trauma patients.

**International Registered Report Identifier (IRRID):**

DERR1-10.2196/14397

## Introduction

### Background

Each year there are thousands of civilian and military limb-threatening lower extremity traumatic injuries [1,2]. Treatment for this is lengthy, with a time and resource burden both for patients and the health system. The outcomes include limb salvage, amputation, or delayed amputation after a failed attempt at reconstruction. It has not yet been established if attempted limb salvage or amputation results in better function, satisfaction, or quality of life for the patient [3,4]. To adequately answer these questions and other important research questions in the management of these patients, a well-defined, valid, reliable, and responsive patient-reported outcome (PRO) instrument is needed. PRO instruments are rating scales that measure concepts of interest (COI) relevant to patients such as the symptoms, appearance, function, and quality of life by asking the patients directly, without interpretation by a clinician or researcher [5].

Numerous PRO instruments have been used in the past to study this patient population, such as the Sickness Impact Profile [6,7] and the Short Musculoskeletal Function Assessment Questionnaire [8]; however, all were developed either for the general population or specific to alternate diseases [9,10]. Despite the previous use of these instruments in lower extremity trauma patients, none were developed specifically for this cohort and, as a result, they lack content specific to this patient population. Therefore, although previous research has been rigorous and well-designed to identify differences in outcomes between the treatment cohorts, the interpretation of these results is limited by inadequate outcome measures used, as they were not designed to address the particular concerns of lower extremity trauma patients.

### Objectives

To ensure that all important COI of these patients are measured and to answer important clinical questions regarding limb reconstruction and amputation, a well-defined PRO instrument designed specifically for lower extremity trauma patients is needed. This instrument should cover common and unique concerns of amputees and limb-reconstruction patients.

**Figure 1:**
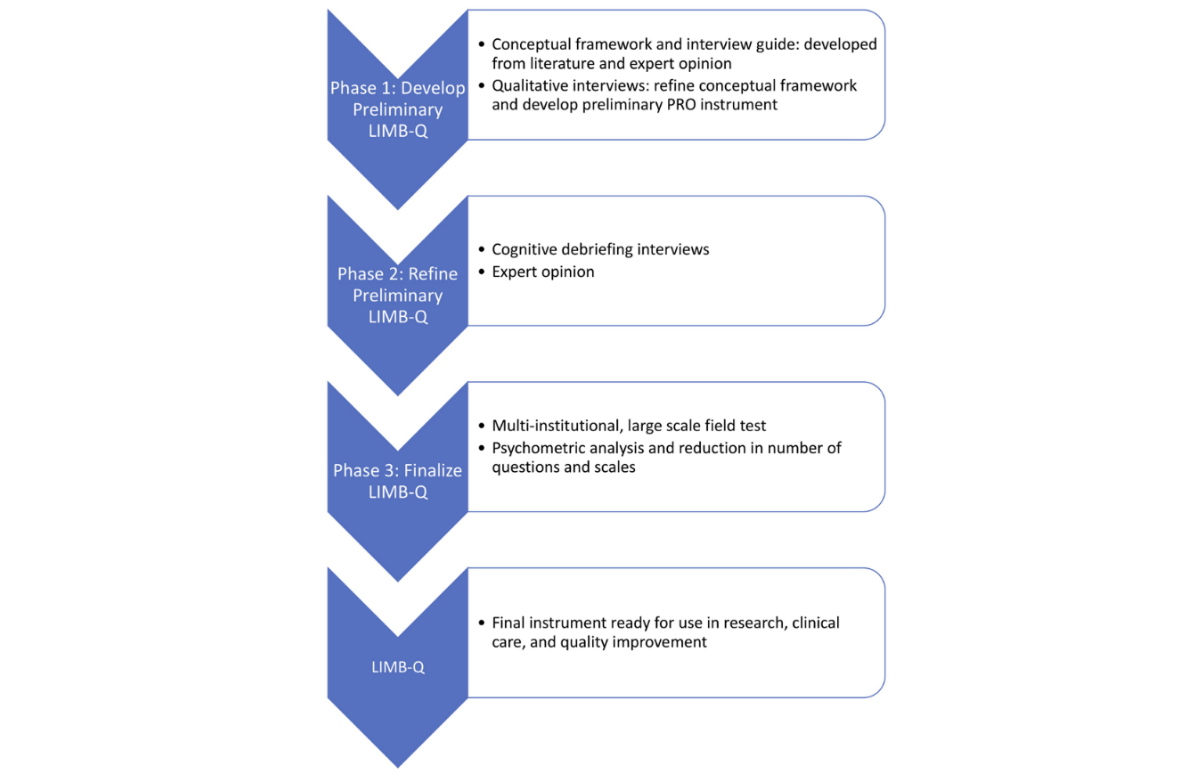
Phases of instrument development. PRO: patient-reported outcome.

The following protocol is the result of an international collaboration whose primary focus is the development and validation of a new PRO instrument, developed specifically for patients after lower extremity trauma, to measure all important COI from the patient’s perspective, applicable to both amputation and reconstruction patients; see Figure 1.

## Methods

### Overview

The aim of this study is to develop a PRO instrument, with a set of independently functioning scales, that captures the multidimensional impact of lower extremity traumatic injuries on patients, useful for clinical practice, quality improvement, and research. Our team follows rigorous, state-of-the-art psychometric methodology for PRO instrument development [5,11-19], utilizing a multiphase mixed-methods approach [20]. Our approach involves 3 main phases of instrument development, which are completed in an iterative and interactive manner. Briefly, the first phase identifies what the instrument should measure through the development of a preliminary conceptual framework from a literature review, followed by qualitative interviews, which are utilized to finalize the conceptual framework and develop the preliminary set of scales. The second phase refines the PRO instrument via cognitive debriefing interviews and solicitation of expert opinion. Finally, the third phase involves a large-scale field test and psychometric evaluation and refinement of items and scales. Adherence to the international guidelines for PRO instrument development ensures that the PRO instrument is scientifically sound (reliable, valid, and responsive). The first 2 phases of instrument development are presented here.

### Conceptual Framework

The conceptual framework describes the COI within the patient group, helping to organize and frame the domains that are of importance to patients, and therefore should be under consideration for inclusion in the PRO instrument [5]. The preliminary conceptual framework was developed from the COI identified in our systematic review of the literature, in addition to expert opinion; see Textbox 1 [9]. The preliminary conceptual framework is then further refined through the qualitative portion of the study. The final conceptual framework maps all COI identified through the qualitative interviews and is used to define the domains within the preliminary set of scales.

**Preliminary conceptual framework domains.**

- Appearance
- Employment
- Physical function
- Prostheses and orthotics
- Psychosocial well-being
- Satisfaction with experience
- Satisfaction with outcome
- Sexual well-being

### Qualitative Study

This is a qualitative study designed to identify the concepts that are most important to lower extremity trauma patients via semistructured qualitative interviews. An interpretive description approach is utilized, with theoretical knowledge from the systematic review and clinical knowledge from the clinical team members, forming a basis for the identification of the COI for this patient group [21].

#### Participants

Inclusion criteria include patients aged 18 years or above, who suffered a lower extremity traumatic injury, distal to the midfemur, resulting in the need for reconstruction or amputation. Patients who do not speak English are excluded. A member of the clinical team recruits patients either face-to-face in the hospital or clinic or over the telephone. Interested participants who are unable to travel to the interview are offered phone interviews.

#### Sampling

Purposeful sampling maximizes participant variability to ensure a diverse group of participants, reflected in demographic variables (age, gender, and race), injury etiology, time since injury, and injury outcome (reconstruction, amputation, or amputation after failed reconstruction). Patients having undergone successful limb salvage, early amputation, and late amputation after failed attempts at reconstruction with varying amounts of time from initial injury will be recruited. Recruitment continues until content saturation is reached, that is, no new concepts are being identified in the interviews [22].

#### Data Collection

**Figure 2:**
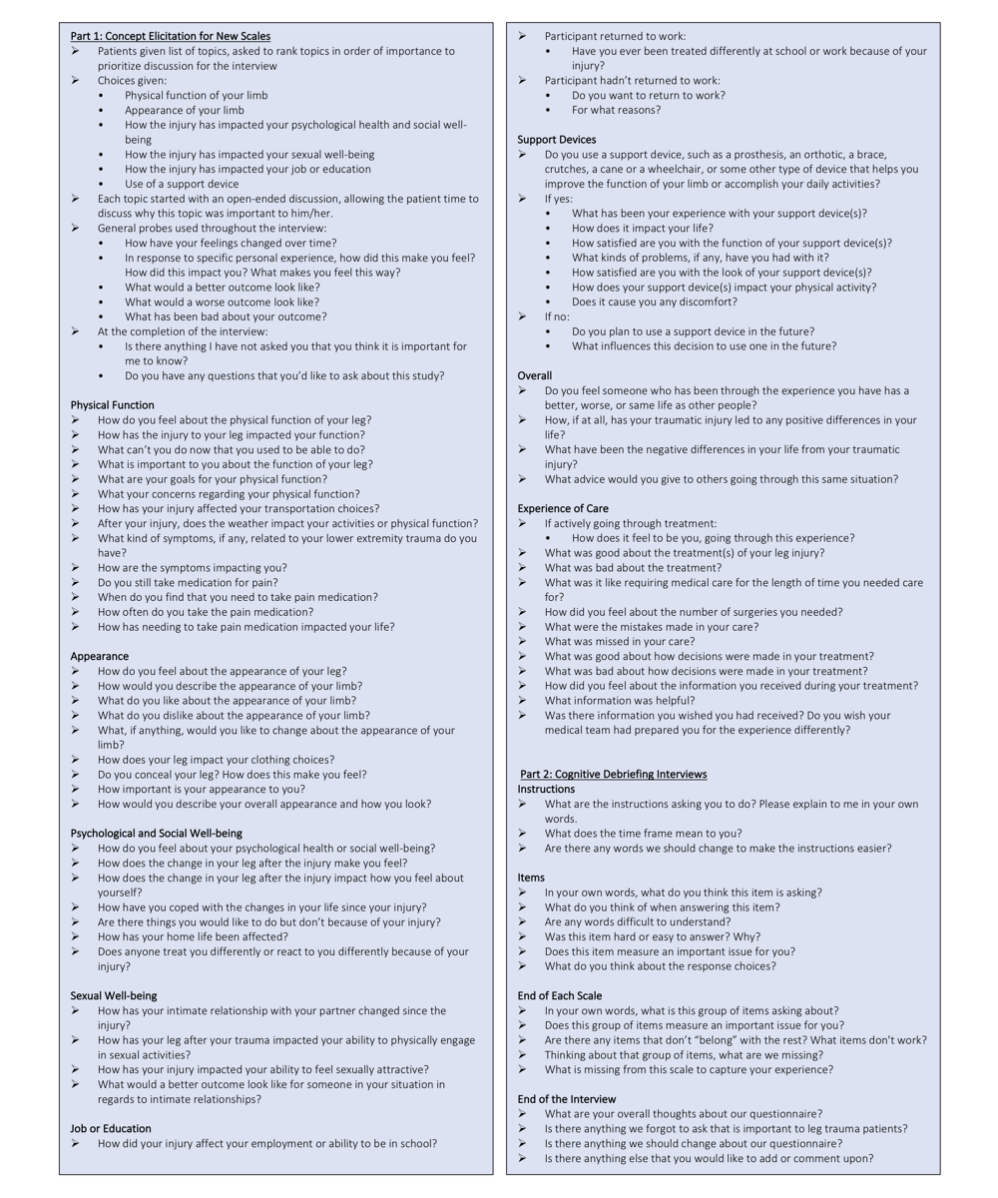
Qualitative interview guide.

Consent is provided before participation. Demographic characteristics include age, gender, and race and clinical characteristics include medical history, drug/tobacco use, injury etiology, injury characteristics, date of injury, and surgical history, in addition to postinjury findings such as return-to-work status and chronic pain medication use. Trained qualitative researchers perform interviews using an interview guide developed to reflect the preliminary conceptual framework to ensure that all previously identified COI are addressed. The interview guide is a working document that is updated throughout the study as new COI are identified; see Figure 2.

#### Data Analysis

Interviews are recorded and transcribed verbatim. Each interview is coded line by line to identify COI, categorized top-down into domains, followed by themes and subthemes. Interviews are coded as a team by a clinical expert (LRM) and qualitative methods expert (AK) to ensure consistency, reliability, and validity in coding. Coding questions requiring additional clinical expertise were discussed with additional clinical experts (MJG and STH) as needed. Constant comparison is used to ensure consistency in coding, allowing for continuous refinement of previously coded data [23]. It must be noted that more than 1 item can be generated from a code if the participant uses different words/phrases to describe their thoughts or experiences. However, if someone uses the same word/phrase repeatedly, we develop 1 item for the concept as we are interested in capturing a unique concept rather than capturing the number of times a subject repeats himself or herself. The domains are used to generate the final conceptual framework, organizing all identified COI.

#### Item Generation

The codes generated in the data analysis process represent an exhaustive list of all relevant COI to lower extremity trauma patients, organized through the lens of the conceptual framework. Each domain within the conceptual framework that is relevant to clinical care or research is utilized to develop a preliminary scale. Coded text with major/minor themes is moved into an Excel spreadsheet (Microsoft Excel 2016) along with the participant’s age, gender, and surgical outcome (reconstruction, amputation, or reconstruction to amputation). Clinical data are utilized to identify the codes that are relevant across patient groups versus the codes that are relevant to only a specific patient group, such as concerns specific to patients with prostheses. Each code is examined in turn to generate a preliminary item pool for scale development. Within each scale, the items are developed from the themes and subthemes, using as many of the participants’ own words as possible. For each scale, instructions and response options are added. Our goal is to keep the scales simple and in line with the published guidelines [24]. Khadka et al demonstrated that rating scales or questionnaires with a complicated question format, with a large number of response categories or with unlabeled categories tended to be dysfunctional and recommended that PRO instruments should have simple question formats and only a few (4-5 at most) response options and that all response options should be labeled [24].

### Refining the Preliminary Scales

The preliminary PRO instrument scales are refined via patient interviews and solicitation of expert opinion in phase II of instrument development, ensuring maximum content clarity and comprehensiveness [19].

#### Cognitive Debriefing Interviews

Semistructured interviews are conducted with lower extremity trauma patients to assess for content and clarity of wording, ensuring the instrument is comprehensive, relevant, and comprehensible. Participants meeting the same inclusion/exclusion criteria stated previously are recruited in a similar manner. Recruitment starts with the initial qualitative interview of participants and then, if needed, is expanded to additional patients meeting the above inclusion criteria, who did not participate in the first round of interviews. Participants are interviewed one-on-one by a trained qualitative interviewer, with the participant going through the scale line by line using the think-aloud technique, explaining out loud how the participant interprets each item [25]. Participants are also asked to comment on the instructions, probed for missing content, and asked to identify problematic items and scales. Consequently, the instrument is prepared for the large-scale field test, combining revisions from the opinions of expert clinicians.

#### Expert Clinical Input

In concurrence with soliciting opinions from patients, the opinions of expert clinicians are solicited. This is to ensure that the PRO instrument captures all key concepts relevant to clinicians or researchers. Expert advice is sought from clinicians, including plastic and orthopedic surgeons, in addition to the physical and occupational therapists, nursing, prosthetists, and limb-loss experts. Experts provide written feedback on the instrument via a written survey.

## Results

Phases I and II of this study have been funded. Phase I of this study has been completed, and phase II began in January 2019 and is expected to be completed in November 2019. Phase III will begin following the completion of phase II.

### Next Steps

#### Pilot Field Test

A pilot field test is carried out before the large-scale international field test. The goal of the pilot field test is 2-fold: first, to identify and address any administrative or logistical difficulties, and second, to perform a preliminary Rasch analysis to identify and address any psychometric issues with LIMB-Q scale performance. Inclusion criteria are similar to those described above. Participants complete the LIMB-Q in addition to providing demographic, injury, and treatment information. Rasch measurement theory (RMT) is used to drive the data analysis of the scales and is described in detail in the following section.

#### International Field Test

A large-scale international field test is the final stage in LIMB-Q development, with the goal to finalize the items and scales of the LIMB-Q, determining scale validity, reliability, and responsiveness. Inclusion criteria are similar to what is described above, with participants recruited from multiple centers in different countries. Centers are selected based on the interest and feasibility of recruitment. In addition to completing the LIMB-Q, participants provide demographic, injury, and treatment information. A small subset of participants complete the LIMB-Q at 2 points, 1 to 2 weeks apart, to allow for an assessment of test-retest reliability. Each scale is analyzed independently so that it may stand alone.

#### Rasch Measurement Theory

In the RMT, the focus is on the relationship between a person’s measurement and his or her probability of responding to an item in a scale [13]. The RMT places individuals onto a *ruler* based on the likelihood of giving a certain answer [26]. The qualitative phase of instrument development allows for the creation sample to fit on the *ruler*, with room to move up or down with treatment, making for scientifically sound and clinically meaningful scales. The RMT analysis identifies the subset of items that are the best indicators of a scale’s concept based on the performance and set of psychometric tests and criteria. Briefly, the psychometric testing of the instrument following the field test will involve several steps. The first will be to assess the psychometric functioning of the items and scales. The thresholds for item response options will be ordered into a hierarchy of items on the scale from the easiest to the most difficult questions. Next, 3 item-fit statistical tests will be employed to determine if the items work well together within a set. These include log residual for item-person interaction, chi-square values for item-trait interaction, and item characteristic curves. Outside of the clinically important items, items not meeting all 3 characteristics will be dropped from the scale. Finally, construct validity is confirmed by comparing the range of the construct measured by the scale with the range experienced by the population. Following these initial steps, the internal consistency, that is, the interrelatedness of items on a scale, will be confirmed with testing for unidimensionality and evaluating the Person Separation Index. In addition, differential item functioning will be evaluated, and items will be reduced accordingly. Items will be reduced that either represent redundancy or overlap on the ruler or are poorly functioning. The steps outlined above will result in a final version of the LIMB-Q and associated scoring table for each scale. Following the development of the finalized scales, construct validity, reliability with internal consistency and test-retest reliability, validity with content, construct and criterion validity, and responsiveness to change will be established.

## Discussion

### Principal Findings

This study is the first to our knowledge to develop a PRO instrument specific to lower extremity trauma patients, applicable to both amputees and limb-salvage patients. A major limitation in previous lower extremity trauma research has been the lack of an appropriate outcome measure that is capable of assessing all COI relevant to both limb-reconstruction patients and amputees [9,10].

The outcome measures currently used to evaluate these patients include functional scales and generic or disease-specific PRO instruments not developed specifically for the lower extremity trauma population. The Lower Extremity Functional Scale [27] or the Locomotor Capabilities Index [28] are functional instruments that provide data deemed important to clinicians and researchers. To evaluate the patient’s perspective, generic PRO instruments, such as the Sickness Impact Profile [6,7] and 36-Item Short Form Health Survey questionnaire [29], or generic musculoskeletal instruments, such as the American Orthopedic Foot and Ankle Score Ankle-Hindfoot and Midfoot Rating Scales [30] and Short Musculoskeletal Function Assessment Questionnaire [8], along with disease-specific instruments not developed for but applied to lower extremity trauma patients, such as the Musculoskeletal Tumor Society [31] and Toronto Extremity Salvage Score [32], are frequently used. Although these instruments are reliable and valid for the general population, or for their respective musculoskeletal disease cohort, they lack the detail needed to measure clinical change in a reliable or meaningful way for these trauma patients [33]. PRO instruments focusing on patients who have undergone only amputation are in use [34]; however, there is no PRO instrument that is designed for all patients with limb-threatening lower extremity traumatic injuries that measures COI relevant to both reconstruction and amputation patient groups.

### Strengths and Limitations

The goal of this protocol is to compete phases I and II of the development of a PRO instrument for lower extremity trauma patients. In future stages, our multidisciplinary team will utilize RMT to finalize the development and validation of LIMB-Q, comprising a comprehensive set of clinically meaningful scales, measuring COI important to patients with limb-threatening lower extremity injuries. The scales will be designed for use in research and direct clinical care, applicable to patients who undergo either limb salvage or amputation. The inclusion of patients at varying time points from injury in the development of this instrument will provide for an agile instrument capable of providing meaningful information to clinicians and patients over the duration of a patient’s treatment course. This instrument will have the capability to measure incremental differences in outcomes important to patients and allow for direct comparison between treatment modalities for severe lower extremity trauma. Clinically, this tool will provide patients with a structured and reliable method for communicating outcomes to providers and will provide accurate and objective feedback to improve clinical care.

### Conclusions

The LIMB-Q will be a novel PRO instrument for use in lower extremity trauma patients, available for use in research and clinical care. Rigorously developed and validated, the LIMB-Q will have the capability to measure COI relevant to lower extremity trauma patients, to help us better care for and understand the needs of these patients with challenging and often devastating injuries.

## Appendix

Multimedia Appendix 1 Peer-review report.

## Abbreviations

COI: concepts of interest
PRO: patient-reported outcome
RMT: Rasch measurement theory
