# Supplementary material for: Development of a Patient-Reported Outcome Instrument for Patients With Severe Lower Extremity Trauma (LIMB-Q): Protocol for a Multiphase Mixed Methods Study
Source: JMIR Res Protoc. 2019 Oct 17;8(10):e14397. doi: 10.2196/14397 (PMC6913330; doi:10.2196/14397)
Supplement: Multimedia Appendix 1 [file resprot_v8i10e14397_app1.pdf]

Duke Clinical Research Institute | FROM THOUGHT LEADERSHIP TO CLINICAL PRACTICE

Notice of Award

Date: October 18, 2017

Principal Investigator(s): Scott Thomas Hollenbeck
Project Title: Development of a PRO Instrument for Use in Severe Lower Extremity Trauma

Dear Dr. Hollenbeck,

Section I – Award Data

Total Direct Budget	$ 15,000

The Fund Code for your project will be 441 1477 and upon IRB approval will be released to your departmental business unit in 2 stages of $7,500 each.

We will review expenses on a quarterly basis to ensure expenses are aligned with how the award was budgeted. We anticipate that expenses will adhere to the budget categories in the proposal submitted. Any major budget deviations (25% or more from intended expenditures) require notification to the Duke MSK faculty advisor.

Consistent with the original RFA announcement, no cost extensions are not allowed and unused funds will be returned to Duke MSK at the end of the award date.

Section II – Terms and Conditions

• Awarded funds must be used to conduct the work proposed. Funds may not be used to supplement federal or private fellowships, training grants, or career development (e.g. K, T, or F) awards.

Funds should not be used for:

- investigator effort
- subcontracts to other institutions
- meals or travel, including to conferences, except as required to collect data
- capital equipment
- office supplies or communication costs, including printing
- professional education or training
- computers or audiovisual equipment
- manuscript preparation and submission, or
- indirect costs
- effort for post-doctoral trainees or fellow on training grant equivalents

• All direct charges to this award must adhere to Duke research spending requirements. We reserve the right to revoke funding in the event it is determined that funds were not spent in accordance with the approved proposal.

Section III – Contacts

Duke MSK Program
Steven George & Kate Sullivan
Email: Steven.George@duke.edu and/or Kathryn.Sullivan@duke.edu

10/18/17

PI Signature (acknowledging acceptance of these terms) Date

Average Scores	
**Significance**	3
**Investigators**	3
**Innovation**	3
**Approach**	3
**Environment**	3
**Overall Impact**	3
**Review Score**	4

NOTE: If a score in the range of 3 -9 is given for any section, a weakness must be listed to give the applicant a reason for the lower score and to allow the applicant to address the weakness in subsequent applications.

Impact	Score	Descriptor	Additional Guidance on Strengths/Weaknesses
High	1	Exceptional	Exceptionally strong with essentially no weaknesses
	2	Outstanding	Extremely strong with negligible weaknesses
	3	Excellent	Very strong with only some minor weaknesses
Medium	4	Very Good	Strong but with numerous minor weaknesses
	5	Good	Strong but with at least one moderate weakness
	6	Satisfactory	Some strengths but also some moderate weaknesses
Low	7	Fair	Some strengths but with at least one major weakness
	8	Marginal	A few strengths and a few major weaknesses
	9	Poor	Very few strengths and numerous major weaknesses

Title: Development of a PRO Instrument for Use in Severe Lower Extremity Trauma
Principal Investigator(s): Scott Hollenbeck

OVERALL IMPACT

Reviewers will provide an overall impact score to reflect their assessment of the likelihood for the project to exert a sustained, powerful influence on the research field(s) involved, in consideration of the following five scored review criteria, and additional review criteria. An application does not need to be strong in all categories to be judged likely to have major scientific impact.

Overall Impact *Write a paragraph summarizing the factors that informed your Overall Impact score.*
While this is a potentially invovative project with potential for future funding, it is ultimately limited by the fact that this project will only be successful if it receives future funding. As a standalone project it is not very exciting and will not lead to any specific research.<br><br>Of equal importance it is concerning that a qualitative researcher was not engaged as a co-investigator. I'm not clear why there is a statistician on the project since there is no data analysis.<br><br>The investigators seek to develop a patient-reported outcome instrument to assess concepts of interest and assess success of reconstructive/limb salvage techniques for patients with severe lower extremity trauma. The study will address an area with need, and the team members are well poised to identify potential study subjects. There are, however, some areas of weakness that could be better defined in the project description.<br><br>The decision to perform an amputation or embark on reconstruction is often aided by clinical impressions and are not supported by national guidelines. Moreover, none of the extant research accounts for either patient preference or patient related outcomes. In this study the investigators seek to develop a validated model to answer this question and aide clinicians when managing these injuries.<br><br>The goals of the project as well as the approach are rationally proposed and thoughtfully articulated. Potential challenges and alternate approaches have been considered. The project is ambitious, but feasible in the timeframe of the grant at the level of support the grant would provide.<br><br>The research team is highly multidisciplinary with both surgery and MSK representation, and includes trainees who are well positioned to benefit from this project, as well external consultants who will provide valuable input. The next steps leading naturally from this proposal are clearly thought through.<br><br>My enthusiasm for this project is very high.<br><br>In this proposal, the authors propose to develop a patient-reported outcome (PRO) measure for quality of life related to lower extremity trauma. This is an unmet need in the field, in which the ultimate outcomes for patients has a critical lack of a patient-reported metric to report satisfaction and quality of life for lower extremity trauma procedures. Such a PRO, which does not currently exist, would provide a patient-centered, objective metric that would be of significant value to patients and physicians forced with difficult decisions regarding dramatic surgeries related to lower extremity trauma (e.g. amputations vs. reconstruction). The proposed team has expertise in PRO development, lower limb kinetics, and reconstructive and orthopedic surgery and involves both experienced faculty and trainees. The proposed approach will provide the critical preliminary data to validate the new PRO and the budget is adequate to support this endeavor, going to support the cost of these interviews. With the data generated by this project, the team will be in an excellent position to apply for future extramural research funds.

SCORED REVIEW CRITERIA

Reviewers will consider each of the five review criteria below in the determination of scientific and technical merit, and give a separate score for each.

1. Significance
**Strengths**<br>• Tries to answer an important question about patient outcomes<br>• Has potential to lead to future funding<br>• Fulfills an as-yet unmet need (no PRO instrument for this patient population)<br>• (From the proposal) Severe lower extremity traumatic injuries are life changing for patients. However, there is a not a clear consensus on the best treatment modality. Treatment options include immediate amputation, delayed amputation after a series of reconstructive attempts, or successful reconstructive limb-salvage. Large prospective trials have shown that successful limb salvage may involve numerous surgeries with a high rate of complications and long-term disability. However, in patients who undergo amputation, the loss of limb is permanent and leads to a life-long reliance on a prosthetic for ambulation. In the setting of modern reconstructive microsurgery, the superior treatment, from the patient perspective, has not yet been established.<br>• Outcomes in lower extremity trauma patients have traditionally been evaluated using clinician-determined measures, evaluating patients based on themes determined to be important by the researcher. However, engaging patients in the assessment is essential, as appearance, function and psychosocial well-being are outcomes best evaluated by patients. There are currently no patient-reported outcome (PRO) instruments that ask patients to report treatment outcomes on their own behalf. PRO instruments are developed using qualitative patient data, and assess concepts of interest (COI) determined to be relevant to patients, such as satisfaction with appearance, body image, function and psychosocial wellbeing.<br>• **The aim for this study is to begin the development of a well-defined, valid, reliable and responsive PRO instrument for patients treated for lower extremity limb-threatening injuries**. The team has designed the project to be the foundation to develop a patient-reported outcome instrument for use in patients with limb-threatening lower extremity injuries to allow for a more patient-focused, evidence-based evaluation of this patient population in research and clinical care. The grant will be used to support initial instrument develop by performing semi-structured qualitative interviews to develop a conceptual framework.<br>• In this proposal, the authors propose to develop a patient-reported outcome (PRO) measure for quality of life related to lower extremity trauma. This is an unmet need in the field, in which the ultimate outcomes for patients has a critical lack of a patient-reported metric to report satisfaction and quality of life for lower extremity trauma procedures. Such a PRO, which does not currently exist, would provide a patient-centered, objective metric that would be of significant value to patients and physicians forced with difficult decisions regarding dramatic surgeries related to lower extremity trauma (e.g. amputations vs. reconstruction).<br>**Weaknesses**<br>• The study does not have strong appeal if it doesn't receive additional funding.

- The scope is very specific and not a lot of potential for broadening.
- How will it be used by clinicians?
- What weigh(s) do patient preferences have in decision-making for limb salvage/reconstruction vs. amputation? Are there other clinical factors that would override these preferences?

2. Investigator(s)

Strengths

- Should have the right clinical team to address the question
- Plans to collaborate with nationally recognized PRO experts (second phase)
- Multi-disciplinary team
- This project is a joint collaboration between the Division of Plastic, Maxillofacial, and Oral Surgery and the Department of Orthopaedic Surgery, with significant involvement by Plastic and Orthopaedic surgical trainees
- The strengths of individual team members are highly complementary and likely to lead to an effective collaboration.
- The external consultants, Drs. Pusic and Klassen, are international leaders in successful development of PRO instruments including the BREAST-Q, FASCE-Q, and others, and will be collaborating with this research team to develop and validate the final instrument
- The proposed team has expertise in PRO development, lower limb kinetics, and reconstructive and orthopedic surgery and involves both experienced faculty and trainees.

Weaknesses

- Unclear why a statistician is part of the team
- Should partner with someone from SSRI who has done qualitative interviews. Having someone with psychometrics background would be useful
- Should include someone with expertise in behavior health and survey research
- Qualitative research analyst (DOCR) not yet identified. This person will be critical to the study

3. Innovation

Strengths

- seems that this is addressing an unmet need in the field
- No instrument currently in use
- (from the proposal) There is currently no measure capturing the patient's voice without influence from a healthcare provider in patients treated for limb-threatening lower extremity injuries. This study will generate the preliminary data for a larger project to complete the development and validation of a PRO instrument for use in lower extremity trauma patients. In the development and validation of the final instrument, our multi-disciplinary team will utilize Rasch Measurement Theory (RMT) to develop this PRO instrument, comprised of a comprehensive set of clinically meaningful scales, measuring

COI important to patients with limb-threatening lower extremity injuries. The scales will be designed for use in research and direct clinical care. Measuring the patient's voice, they will allow PROs to be a component of lower extremity trauma research.

- This proposal addresses an unmet need in the field, in which the ultimate outcomes for patients has a critical lack of a patient-reported metric to report satisfaction and quality of life for lower extremity trauma procedures. No such PRO currently exists.

Weaknesses

- By itself this study won't push the field further

4. Approach

Strengths

- Have thought through their strategy for this project and how it will lead to future work
- Using previously-described methods, which have been successfully adopted
- The study will be designed to capture all the distinct health domains relevant to this population of patients. A qualitative research method will be used, guided by state-of-the-art PRO instrument development methods to maximize both the clinical meaning and scientific quality. Qualitative interviews of a heterogeneous sample of 40 patients will be audio- recorded, transcribed and then coded line-by-line to identify COI. These COI will guide the development of a conceptual framework and a set of scales. The scales will contain items that map out the clinical hierarchy for each COI. It is presumed that some COI will be relevant to all patients and some may be relevant to only a subgroup of patients, such as those with a successful limb-salvage. If needed, given the heterogeneity of this patient population, future studies will collect additional qualitative interview data and subsequently field test this PRO instrument, allowing for item reduction and a test of psychometric properties (e.g., reliability, validity and responsiveness to change). Our long-term goal is to make this instrument available for widespread use in research and clinical care.
- This is a qualitative study with the following steps: 1) semi-structured patient interviews to collect data, and 2) development of a hypothesized conceptual framework and set of scales with items reflective of COI identified from qualitative data gathered in step 1. Future research will include steps: 3) cognitive interviews refining the preliminary set of scales and items to ensure they contain maximum content validity and are easy to comprehend, 4) expert opinion refining the scales, and 5) field testing of the instrument to allow for item reduction and testing of psychometric properties.
- Patients will be recruited for qualitative interviews who are age 18 years and older and are receiving or have received care at Duke University for a limb-threatening lower extremity injury. The patient population has been clearly defined, including the level of amputation, associated injuries, and exclusion criteria (e.g. spinal cord deficit, non-ambulatory state or amputation prior to traumatic injury, psychiatric disorder, etc).
- Plan for conduct and analysis for the qualitative interviews is clearly described.
- The proposed approach will provide the critical preliminary data to validate the new PRO and the budget is adequate to support this endeavor, going to support the cost of these interviews. With the data generated by this project, the team will be in an excellent position to apply for future extramural research funds.

Weaknesses

- The applicants mention wanting to interview 40 people but no mention of how many such procedures are performed so hard to evaluate whether this is feasible or not.
- Unclear as to what timepoint in the clinical course that potential study subjects will be contacted to participate. Would variation in this potentially alter the study results?
- The sample size of 40 survey participants may be difficult to achieve; however the PI presents several alternative recruitment strategies to overcome this challenge. Given that recruitment may be an important and possible issue, the team should consider many methods of patient recruitment from study outset.

5. Environment

Strengths

- Good Clinical environment
- The preliminary data the team will generate in this project has high likelihood of success in forming the foundation for a externally funded research grant to complete the patient-reported outcome instrument development and validation. The PI plans to complete this second stage with Drs. Andrea Pusic, MD at Memorial Sloan Kettering Cancer Center and Anne Klassen, DPhil at McMaster University. Drs. Pusic and Klassen are international experts in the development of patient-reported outcome instruments and have collaborated with the applicants on multiple previous research endeavors.
- Ideal collaborative effort including both reconstructive and orthopedic surgeons.

Weaknesses

- Would have liked to seen more partnership with individuals doing qualitative research across campus. Not sure if DOCR is really the right target for this.
- Is there enough clinical volume to accrue 40 patients to the study within 8 months?

FOT

Striving to influence and enhance orthopedic traumatology through education, mentorship and research founded on firm clinical grounds

August 8, 2018

Dear Dr. Gage,

Congratulations! We are pleased to inform you that your grant proposal entitled*: Developmentof a Patient-reported Outcome Instrument for Severe Lower Extremity Trauma:Cognitive Debriefing Interviews (FOT GRANT ID#5018-2)* has been approved for funding in the amount of $19,986.00. Please be advised of the stipulation that the grant award does not cover any indirect costs. Please identify where the funds are to be sent and how the funds are to be made payable. This information can be sent via email to : administrator@fotnorthamerica.org. This grant is made possible by funding from the Foundation for Orthopedic Trauma. Any citation regarding the research done with this grant and funded project must identify the Foundation for Orthopedic Trauma as a funding source.

The Foundation for Orthopedic Trauma requires a progress/final report at 12 months which should an accounting of your expenditures. A final report will be due within 60 days of completion of the grant. You are encouraged to submit any resulting manuscript to an appropriate refereed journal such as the Journal of Orthopaedic Trauma. An accepted manuscript should identify Foundation for Orthopedic Trauma as a funding source.

Please review and signed the attached research grant policies and guidelines and return to the above email address as well.

Sincerely,

Jaimo Ahn, MD
Research Committee Chair, Foundation for Orthopedic Trauma

: Attachments-FOT Research Grant policies/guidelines

Foundation for Orthopedic Trauma PO Box 98, Pearl River, NY 10965
Phone:212-305-6392/Fax:212-342-1749
